# Commentary: GARLH Family Proteins Stabilize GABA_A_ Receptors at Synapses

**DOI:** 10.3389/fnmol.2017.00169

**Published:** 2017-05-29

**Authors:** Chang-Hoon Cho

**Affiliations:** School of Public Health, Korea UniversitySeoul, South Korea

**Keywords:** GABA_A_ receptor, GARLH4, GARLH3, inhibitory synapse, Neuroligin-2, gephyrin

GABAergic inhibition at symmetric synapses balances excitation, modulates the spike timing of various neurons, controls oscillatory network activities, and manages emerging properties in diverse neuronal circuits, which establish the basis for cognitive functions and behaviors (Klausberger and Somogyi, 2008; Buzsáki and Wang, 2012). To perform these diverse functions some interneurons form synapses exclusively on the dendrites of other neurons while others target the soma (Maccaferri, 2005; Freund and Katona, 2007). Therefore, it is reasonable to think that GABAergic synapses are heterogeneous, composed by recruiting various subunits of GABA_A_ receptors and their interacting proteins to form macromolecular complexes at the inhibitory synapses (Mann and Paulsen, 2007). Different types of GABAergic neurons also undergo dynamic changes during the early developmental period as well as in synaptic size and morphology, and these interneurons form or eliminate inhibitory synapses (Vogels et al., 2013; Antonelli et al., 2014; Flores and Méndez, 2014; Zacchi et al., 2014; Lu et al., 2017). Altered expressions and/or dysfunctions of several key interacting proteins of GABA_A_ receptors have been associated with schizophrenia, autism, epilepsy, mood disorders, Alzheimer's disease, and other neurological disorders caused by mutations, copy number variations, and single nucleotide polymorphisms (Ko et al., 2015).

Recently several studies reported growing numbers of proteins involved in inhibitory synapses (Kang et al., 2014; Loh et al., 2016; Nakamura et al., 2016; Uezu et al., 2016; Yamasaki et al., 2017). These molecules include ion channels, GPCRs, transporters, cytoskeletal proteins, adhesion proteins, signaling molecules including kinases and phosphatases, and ubiquitination-related proteins. Although it seems redundant to search for interacting proteins using different but closely related “bait” proteins, beside GABA_A_R subunits and their core binding proteins (e.g., gephyrin and neuroligin-2), proteins found at GABAergic synapses from these studies are distinct ones that lead us to speculate about their functional implications. First, by using transgenic mice with His6-FLAG-YFP tagging to Neuroligin-2, 76 proteins were identified as neuroligin-2 interacting proteins (Kang et al., 2014). Second, by using HRP-tagging to Neuroligin-2 and SLITRK3, 44 inhibitory synapse-specific proteins (vs. excitatory synapse-specific ones) including two synaptic orphan molecules (CSMD1/3 and CDH20) and MDGA2, were identified (Loh et al., 2016). Third, by using the Bio-ID tagging to gephyrin, 181 proteins were identified at the inhibitory postsynaptic density, which include ARHGEF9/Collybistin, Mena and Evl, IQSEC3, Px-RICS and two related proteins with unknown functions, InSyn1 and InSyn2 (Uezu et al., 2016). Interestingly, gephyrin, collybistin, and InSyn1 serve as three independent hub proteins that interact with different proteins at the inhibitory synapses (Uezu et al., 2016). Fourth, by using GFP and Myc epitope tagging of the α2 subunit of GABA_A_ receptors in transgenic mice, 174 proteins were identified as interacting proteins including cullin1, ephexin, KTDP12, mitofusin2, mGluR5, PAK7, and RAP5A (Nakamura et al., 2016).

Now, Yamasaki et al. used mass spectrometry to identify the GARLH protein family, GARLH4 (GABA_A_ receptor regulatory Lhfpl4) and GARLH3, as putative auxiliary subunits of GABA_A_Rs (Yamasaki et al., 2017). The search for these proteins of GABA_A_Rs was motivated on the report that the amplitude, but not the frequency, of miniature inhibitory postsynaptic currents (mIPSCs) was modestly decreased when gephyrin, a well-known GABA_A_R associated protein, was eliminated in neurons (Lévi et al., 2004). Therefore, it is speculated that gephyrin-independent, novel GABA_A_R-interacting proteins can be functioning at inhibitory synapses. GARLH4, in addition to the GABA_A_R γ2 subunit and neuroligin-2, is shown to be required to reconstitute the large GABA_A_R complex (720 kDa). Although direct interaction between GARLH4 and the GABA_A_R γ2 subunit is yet to be shown, Yamasaki et al., showed that GARLH4 stabilizes γ2-containing GABA_A_ receptors at inhibitory synapses and connects γ2 subunit and neuroligin-2. By using a transgenic mouse line (Gabra6-Cre), where gabrg2 was specifically deleted in cerebellar granule cells, it was found that levels of GARLH4 and neuroligin-2 were reduced. This indicates that γ2-containing GABA_A_ receptors stabilize GARLH4 protein expression in the cerebellum. In addition, the specific shRNA-mediated silencing of GARLH4 reduced clustering of γ2 GABA_A_R subunit, gephyrin, and neuroligin-2. In cultured hippocampal neurons, silencing of GARLH4 reduced the frequency, but not the amplitude, of mIPSC without affecting its decay kinetics compared to the one of mIPSCs of control neurons. In experiments using sgRNA-mediated deletion of GARLH4 in Cas9 knockin mice, the frequency, but not the amplitude of mIPSCs is decreased in acute hippocampal slice preparations as seen with cultured neurons. However, GARLH4 did not modulate the surface expression or sensitivity of agonists (GABA and THIP) and antagonist (picrotoxin) of α1β2γ2 GABA_A_Rs heterogeneously expressed in *Xenopus* oocytes. It is yet to be examined if the effects of allosteric modulators (e.g., benzodiazepines and neurosteroids) or other various combinations of GABA_A_R subunits (e.g., α1β3γ2 or α6β2γ2) are influenced in the presence of GARLH4.

GARLH4 and GARLH3 are likely to be four transmembrane proteins with both termini facing the cytosol. Therefore, there are possibilities of post-translational modification(s) or activity-dependent protein-protein interaction(s) of these two molecules. In addition, both have a putative ubiquitination residue (K9) at the N-terminal cytoplasmic domain (www.phosphositeplus.org). Since GARLH4 stabilizes GABA_A_ receptors at the synapse, it will be intriguing to see if and/or how GARLH4 plays a role in the activity-dependent plasticity of GABAergic synapses (Antonelli et al., 2014). GARLH4 and GARLH3 are particularly interesting because unlike most other GABA_A_R binding proteins, their expressions are region-specific (GARLH3-cerebellum and GARLH4- cerebellum and hippocampus). Thus, it might be suitable to elucidate the functions of these molecules by knocking out these molecules in a tissue specific manner. In addition, since GARLH3 has been implicated in primary glioblastoma, it may be interesting to see if and/or how GARLH3 displays the unknown contribution of inhibitory synapses to glioma or its *de novo* expression in glioma occurs without affecting GABAergic synaptic transmission (Milinkovic et al., 2013). Lastly, how does this GARLH4-GABA_A_-R association play a role early during development, in neurons with elevated [Cl−]_i_ (e.g., DRG neurons), or in diseased states when GABAergic activation is excitatory (e.g., epilepsy)? Now we have taken another exciting step in dissecting the specific roles of each molecule at the particular inhibitory synapses between unique combinations of neurons.

## Author contributions

The author confirms being the sole contributor of this work and approved it for publication.

### Conflict of interest statement

The author declares that the research was conducted in the absence of any commercial or financial relationships that could be construed as a potential conflict of interest. The reviewer TJ and handling Editor declared their shared affiliation, and the handling Editor states that the process nevertheless met the standards of a fair and objective review.

## References

[B1] Antonelli R. Pizzarelli R. Pedroni A. Fritschy J. M. Del Sal G. Cherubini E.. (2014). Pin1-dependent signalling negatively affects GABAergic transmission by modulating neuroligin2/gephyrin interaction. Nat. Commun. 5:5066. 10.1038/ncomms6066 25297980 PMC4197815

[B2] Buzsáki G. Wang X. J. (2012). Mechanisms of gamma oscillations. Annu. Rev. Neurosci. 35, 203–225. 10.1146/annurev-neuro-062111-150444 22443509 PMC4049541

[B3] Flores C. E. Méndez P. (2014). Shaping inhibition: activity dependent structural plasticity of GABAergic synapses. Front. Cell. Neurosci. 8:327. 10.3389/fncel.2014.00327 25386117 PMC4209871

[B4] Freund T. F. Katona I. (2007). Perisomatic inhibition. Neuron 56, 33–42. 10.1016/j.neuron.2007.09.012 17920013

[B5] Kang Y. Ge Y. Cassidy R. M. Lam V. Luo L. Moon K. M.. (2014). A combined transgenic proteomic analysis and regulated trafficking of neuroligin-2. J. Biol. Chem. 289, 29350–29364. 10.1074/jbc.M114.549279 25190809 PMC4200284

[B6] Klausberger T. Somogyi P. (2008). Neuronal diversity and temporal dynamics: the unity of hippocampal circuit operations. Science 321, 53–57. 10.1126/science.1149381 18599766 PMC4487503

[B7] Ko J. Choii G. Um J. W. (2015). The balancing act of GABAergic synapse organizers. Trends Mol. Med. 21, 256–268. 10.1016/j.molmed.2015.01.004 25824541

[B8] Lévi S. Logan S. M. Tovar K. R. Craig A. M. (2004). Gephyrin is critical for glycine receptor clustering but not for the formation of functional GABAergic synapses in hippocampal neurons. J. Neurosci. 24, 207–217. 10.1523/JNEUROSCI.1661-03.2004 14715953 PMC6729579

[B9] Loh K. H. Stawski P. S. Draycott A. S. Udeshi N. D. Lehrman E. K. Wilton D. K.. (2016). Proteomic analysis of unbounded cellular compartments: synaptic clefts. Cell 166, 1295–1307.e21. 10.1016/j.cell.2016.07.041 27565350 PMC5167540

[B10] Lu W. Bromley-Coolidge S. Li J. (2017). Regulation of GABAergic synapse development by postsynaptic membrane proteins. Brain Res. Bull. 129, 30–42. 10.1016/j.brainresbull.2016.07.004 27453545 PMC5253122

[B11] Maccaferri G. (2005). Stratum oriens horizontal interneurone diversity and hippocampal network dynamics. J. Physiol. 562, 73–80. 10.1113/jphysiol.2004.077081 15498801 PMC1665470

[B12] Mann E. O. Paulsen O. (2007). Role of GABAergic inhibition in hippocampal network oscillations. Trends Neurosci. 30, 343–349. 10.1016/j.tins.2007.05.003 17532059

[B13] Milinkovic V. Bankovic J. Rakic M. Stankovic T. Skender-Gazibara M. Ruzdijic S.. (2013). Identification of novel genetic alterations in samples of malignant glioma patients. PLoS ONE 8:e82108. 10.1371/journal.pone.0082108 24358143 PMC3864906

[B14] Nakamura Y. Morrow D. H. Modgil A. Huyghe D. Deeb T. Z. Lumb M. J.. (2016). Proteomic characterization of inhibitory synapses using a novel pHluorin-tagged γ-Aminobutyric acid receptor, Type A (GABA_A_), α2 Subunit Knock-in Mouse. J. Biol. Chem. 291, 12394–12407. 10.1074/jbc.M116.724443 27044742 PMC4933285

[B15] Uezu A. Kanak D. J. Bradshaw T. W. Soderblom E. J. Catavero C. M. Burette A. C.. (2016). Identification of an elaborate complex mediating postsynaptic inhibition. Science 353, 1123–1129. 10.1126/science.aag0821 27609886 PMC5432043

[B16] Vogels T. P. Froemke R. C. Doyon N. Gilson M. Haas J. S. Liu R.. (2013). Inhibitory synaptic plasticity: spike timing-dependence and putative network function. Front. Neural Circuits. 7:119. 10.3389/fncir.2013.00119 23882186 PMC3714539

[B17] Yamasaki T. Hoyos-Ramirez E. Martenson J. S. Morimoto-Tomita M. Tomita S. (2017). GARLH Family Proteins Stabilize GABA(A) Receptors at Synapses. Neuron. 93, 1138–1152.e6. 10.1016/j.neuron.2017.02.023 28279354 PMC5347473

[B18] Zacchi P. Antonelli R. Cherubini E. (2014). Gephyrin phosphorylation in the functional organization and plasticity of GABAergic synapses. Front. Cell. Neurosci. 8:103. 10.3389/fncel.2014.00103 24782709 PMC3988358

